# Supplementary material for: Differential Exoproteome and Biochemical Characterisation of Neoparamoeba perurans
Source: Microorganisms. 2021 Jun 9;9(6):1258. doi: 10.3390/microorganisms9061258 (PMC8226569; doi:10.3390/microorganisms9061258)
Supplement: Supplementary file 1 [file microorganisms-09-01258-s001.zip › microorganisms-1185006-supplementary.pdf]

Differential exoproteome and biochemical characterisation of *Neoparamoeba perurans*

Kerrie Ní Dhufaigh[a*], Eugene Dillon[b], Natasha Botwright[c], Ian O'Connor[a], Eugene MacCarthy[a], Orla Slattery[d]

[a]Marine and Freshwater Research Centre, Galway-Mayo Institute of Technology, Co. Galway, Ireland

[b]Conway Institute, University College Dublin, Co. Dublin, Ireland

[c]CSIRO Agriculture and Food, Livestock & Aquaculture, Queensland Biosciences Precinct, 306 Carmody Road, Brisbane, Queensland 4067, Australia

[d]Department of Biopharmaceutical and Medical Science, Galway-Mayo Institute of Technology, Co. Galway, Ireland

*Corresponding author. Marine and Freshwater Research Centre, Galway-Mayo Institute of Technology, Co. Galway, Ireland

Email: kerrie.nidhufaigh@research.gmit.ie

Table S1: Bacterial reference proteome downloaded on the 8th of May 2020 from UniProt

Proteome ID	Organism	Organism ID	Protein count	BUSCO	CPD	Genome representation (RefSeq)
UP000198862	Pseudoalteromonas denitrificans DSM 6059 (Strain: DSM 6059)	1123010	5332	C:98.7%[S:98.2%,D:0.4%],F:0.4%,M:0.9%,n:452	Close to Standard	full
UP000033452	Pseudoalteromonas rubra (Strain: S2471)	43658	4390	C:98.7%[S:98.5%,D:0.2%],F:0%,M:1.3%,n:452	Standard	full
UP000033511	Pseudoalteromonas piscicida (Strain: S2040)	43662	4134	C:97.1%[S:96.7%,D:0.4%],F:0.2%,M:2.7%,n:452	Close to Standard	full
UP000179786	Pseudoalteromonas amylolytica (Strain: JW1)	1859457	4015	C:99.3%[S:98.9%,D:0.4%],F:0.4%,M:0.2%,n:452	Standard	full
UP000030341	Pseudoalteromonas piratica (Strain: OCN003)	1348114	3997	C:99.8%[S:99.3%,D:0.4%],F:0%,M:0.2%,n:452	Standard	full
UP000033664	Pseudoalteromonas ruthenica (Strain: S3137)	151081	3470	C:98.5%[S:98%,D:0.4%],F:0.2%,M:1.3%,n:452	Standard	full
UP000061457	Pseudoalteromonas phenolica (Strain: KCTC 12086)	161398	4164	C:98.9%[S:98.2%,D:0.7%],F:0.4%,M:0.7%,n:452	Standard	full
UP000006843	Pseudoalteromonas haloplanktis (strain TAC 125) (Strain: TAC 125)	326442	3484	C:99.3%[S:98.9%,D:0.4%],F:0.2%,M:0.4%,n:452	Standard	full
UP000016487	Pseudoalteromonas citrea DSM 8771 (Strain: DSM 8771)	1117314	4457	C:99.6%[S:99.3%,D:0.2%],F:0.2%,M:0.2%,n:452	Standard	full
UP000194841	Pseudoalteromonas ulvae	107327	3964	C:99.3%[S:98.9%,D:0.4%],F:0%,M:0.7%,n:452	Standard	full
UP000076643	Pseudoalteromonas luteoviolacea DSM 6061 (Strain: DSM 6061)	1365250	5008	C:99.6%[S:98.9%,D:0.7%],F:0%,M:0.4%,n:452	Standard	full
UP000007127	Thalassospira xiamenensis M-5 = DSM 17429 (Strain: M-5)	1123366	4340	C:98.2%[S:98.2%,D:0%],F:0.5%,M:1.4%,n:221	Standard	full
UP000252266	Thalassospira xiamenensis (Strain: IB13)	220697	4080	C:96.4%[S:96.4%,D:0%],F:0.9%,M:2.7%,n:221	Standard	full
UP000253064	Thalassospira xiamenensis (Strain: S27-11)	220697	4028	C:97.3%[S:97.3%,D:0%],F:0.5%,M:2.3%,n:221	Standard	full

Proteome ID	Organism	Organism ID	Protein count	BUSCO	CPD	Genome representation (RefSeq)
UP000219068	Thalassospira xiamenensis (Strain: USBA 78)	220697	5070	C:98.2%[S:95.9%,D:2.3%],F:0.5%,M:1.4%,n:221	Outlier	full
UP000007463	Fluviicola taffensis (strain DSM 16823 / NCIMB 13979 / RW262) (Strain: DSM 16823 / RW262 / RW262)	755732	4016	C:97.1%[S:96.6%,D:0.5%],F:1.6%,M:1.4%,n:443	Close to Standard	full
UP000000584	Vibrio cholerae serotype O1 (strain ATCC 39315 / El Tor Inaba N16961) (Strain: ATCC 39315 / El Tor Inaba N16961)	243277	3782	C:98.9%[S:98.5%,D:0.4%],F:0%,M:1.1%,n:452	Standard	full
UP000000537	Aliivibrio fischeri (strain ATCC 700601 / ES114) (Vibrio fischeri) (Strain: ATCC 700601 / ES114)	312309	3813	C:99.3%[S:99.1%,D:0.2%],F:0.7%,M:0%,n:452	Standard	full
UP000232179	Vibrio sp. HA2012 (Strain: HA2012)	1971595	3382	C:99.8%[S:99.6%,D:0.2%],F:0%,M:0.2%,n:452	Standard	full
UP000235640	Vibrio sp. 10N.286.49.C2 (Strain: 10N.286.49.C2)	1880856	4338	C:99.8%[S:98.7%,D:1.1%],F:0%,M:0.2%,n:452	Standard	full
UP000009100	Vibrio tasmaniensis (strain LGP32) (Vibrio splendidus (strain Mel32)) (Strain: LGP32)	575788	4420	C:99.1%[S:97.6%,D:1.5%],F:0.9%,M:0%,n:452	Standard	full
UP000094165	Vibrio genomosp. F6 str. FF-238 (Strain: FF-238)	1191298	3650	C:98.7%[S:98.5%,D:0.2%],F:0.4%,M:0.9%,n:452	Standard	full
UP000094070	Vibrio rumoiensis 1S-45 (Strain: 1S-45)	1188252	2981	C:99.1%[S:99.1%,D:0%],F:0%,M:0.9%,n:452	Close to Standard	full
UP000002943	Vibrio caribbeanicus ATCC BAA-2122 (Strain: ATCC BAA-2122)	796620	4023	C:99.3%[S:99.3%,D:0%],F:0.2%,M:0.4%,n:452	Standard	full
UP000184608	Vibrio aerogenes CECT 7868 (Strain: CECT 7868)	1216006	4565	C:99.3%[S:99.1%,D:0.2%],F:0.7%,M:0%,n:452	Standard	full
UP000281112	Vibrio sp. LJC006 (Strain: LJC006)	2487322	4277	C:100%[S:99.3%,D:0.7%],F:0%,M:0%,n:452	Standard	full
UP000016567	Vibrio azureus NBRC 104587 (Strain: NBRC 104587)	1219077	4147	C:99.6%[S:98.9%,D:0.7%],F:0.4%,M:0%,n:452	Standard	full
UP000002493	Vibrio parahaemolyticus serotype O3:K6 (strain RIMD 2210633) (Strain: RIMD 2210633)	223926	4821	C:99.6%[S:98.7%,D:0.9%],F:0.2%,M:0.2%,n:452	Standard	full
UP000193432	Vibrio sp. qd031 (Strain: qd031)	1603038	3553	C:98.5%[S:98%,D:0.4%],F:0%,M:1.5%,n:452	Standard	full

Proteome ID	Organism	Organism ID	Protein count	BUSCO	CPD	Genome representation (RefSeq)
UP000269041	Vibrio pectenicida (Strain: CAIM 594)	62763	3823	C:99.1%[S:94.7%,D:4.4%],F:0%,M:0.9%,n:452	Standard	full
UP000037515	Vibrio nereis (Strain: DSM 19584)	693	3660	C:100%[S:99.6%,D:0.4%],F:0%,M:0%,n:452	Standard	full
UP000189475	Vibrio palustris (Strain: CECT 9027)	1918946	3328	C:99.6%[S:98.2%,D:1.3%],F:0.2%,M:0.2%,n:452	Standard	full
UP000029994	Vibrio navarrensis (Strain: ATCC 51183)	29495	3643	C:99.8%[S:99.1%,D:0.7%],F:0%,M:0.2%,n:452	Standard	full
UP000016895	Vibrio nigripulchritudo (Strain: SnF1)	28173	5540	C:100%[S:99.8%,D:0.2%],F:0%,M:0%,n:452	Standard	full
UP000003627	Vibrio sp. (strain N418) (Strain: N418)	701176	4028	C:99.6%[S:98.2%,D:1.3%],F:0.4%,M:0%,n:452	Standard	full
UP000198854	Vibrio xiamenensis (Strain: CGMCC 1.10228)	861298	4859	C:100%[S:98.9%,D:1.1%],F:0%,M:0%,n:452	Standard	full

Table S2: Amoebozoa reference proteome downloaded on the 8th of May 2020 from UniProt

Proteome ID	Organism	Organism ID	Protein count	BUSCO	CPD	Genome representation (RefSeq)
UP000001064	Dictyostelium purpureum (Slime mold) (Strain: QSDP1)	5786	12347	C:92.1%[S:89.1%,D:3%],F:2%,M:5.9%,n:303	Standard	full
UP000002195	Dictyostelium discoideum (Slime mold) (Strain: AX4)	44689	12746	C:96%[S:92.4%,D:3.6%],F:0.7%,M:3.3%,n:303	Standard	full
UP000001396	Polysphondylium pallidum (strain ATCC 26659 / Pp 5 / PN500) (Heterostelium pallidum) (Strain: ATCC 26659 / Pp 5 / PN500)	670386	12351	C:92.1%[S:92.1%,D:0%],F:3.6%,M:4.3%,n:303	Standard	full
UP000001926	Entamoeba histolytica (Strain: ATCC 30459 / HM-1:IMSS)	5759	7959	C:65.7%[S:54.1%,D:11.6%],F:6.9%,M:27.4%,n:303	Standard	full
UP000241769	Planoprotostelium fungivorum (Strain: Jena)	1890364	16856	C:94.4%[S:87.5%,D:6.9%],F:2%,M:3.6%,n:303	Outlier	full
UP000014680	Entamoeba invadens IP1 (Strain: IP1)	370355	9857	C:53.8%[S:49.5%,D:4.3%],F:4.3%,M:41.9%,n:303	Close to Standard	full
UP000076078	Tieghemostelium lacteum (Strain: TK)	361077	10208	C:93.4%[S:92.4%,D:1%],F:2.3%,M:4.3%,n:303	Outlier	full
UP000011083	Acanthamoeba castellanii str. Neff (Strain: Neff)	1257118	14939	C:81.5%[S:77.6%,D:4%],F:5.9%,M:12.5%,n:303	Close to Standard	full
UP000007797	Cavenderia fasciculata (strain SH3) (Slime mold) (Dictyostelium fasciculatum) (Strain: SH3)	1054147	12152	C:92.1%[S:90.4%,D:1.7%],F:2.3%,M:5.6%,n:303	Standard	full

Table S3: Paramoeba taxonomy search on UniProt KB downloaded on 8th of May 2020.

Organism	Total protein Count	Taxon ID
Candidatus Syngnamydia salmonis	9	504270
Paramoeba aestuarina	1	180227
Paramoeba aparasomata	39	2583407
Paramoeba branchiphila	1	308475
Paramoeba eilhardi	1	200891
Paramoeba invadens	1	1321612
Paramoeba karteshi	2	2583406
Paramoeba pemaquidensis	62	180228
Paramoeba perurans	2	437603
Perkinsela sp. CCAP 1560/4	4883	1314962

Table S4: Identification of bacterial proteins from attenuated and virulent *Neoparamoeba perurans* exoproteomes characterised by a two-way Students *t*- test in Perseus. Statistically significant differential proteins are represented with a + sign based on q-value.

**Gene**	**Protein IDs**	**Fasta headers**	**Organism**	**Mol. weight [kDa]**	**Fold change[a]**	**Att. Peptides[b]**	**Vir. Peptides[b]**	**T-test[c]**
ST37_14230	A0A1X1MR06	ABC transporter substrate-binding protein	Vibrio sp. qd031	47.26	-3.18528	6	0	+
VS_0075	B7VH98	Periplasmic dipeptide transport protein	Vibrio tasmaniensis (strain LGP32)	57.321	-5.41027	22	2	+
VS_2212; BCU70_21010	B7VI06; A0A2N7D1D1	Extracellular solute-binding protein, family 7; C4-dicarboxylate ABC transporter	Vibrio tasmaniensis (strain LGP32); Vibrio sp. 10N.286.49.C2	36.945	-5.87885	19	8	+
VS_II0220	B7VQI9	Maltodextrin-binding protein	Vibrio tasmaniensis (strain LGP32	42.386	-4.90439	13	4	+
VS_0757; ushA	B7VKH8; A0A0M0HJC5	5-nucleotidase	Vibrio tasmaniensis (strain LGP32); Vibrio nereis	62.392	-0.414253	7	9	+
PSHAa0782	Q3IDX3	Flagellin	Pseudoalteromonas haloplanktis (strain TAC 125)	28.577	-0.683505	13	15	+
PP2015; BET10_08905	A0A0S2JZH9; A0A1S1MTX5	Flagellar hook protein FlgE	Pseudoalteromonas phenolica; Pseudoalteromonas amylolytica	47.109	-4.78254	8	6	+
BET10_13780; PSHAa059	A0A1S1MX56; Q3ILL3	Phosphate-binding protein ; Putative phosphate ABC transporter	Pseudoalteromonas amylolytica; Pseudoalteromonas haloplanktis (strain TAC 125)	35.016	-4.4918	9	0	+
VS_0355	B7VIC7	Immunogenic protein	Vibrio tasmaniensis (strain LGP32)	34.436	-5.20998	11	1	+

Gene	Protein IDs	Fasta headers	Organism	Mol. weight [kDa]	Fold change[a]	Att. Peptides[b]	Vir. Peptides[b]	T-test[c]
VS_1055; EA26_05975	B7VM14	Lysine-arginine-ornithine-binding periplasmic protein; Nickel transporter	Vibrio tasmaniensis (strain LGP32); Vibrio navarrensis	28.109	-6.24674	11	0	+
ahpCB	Q3IE21	Peroxiredoxin 2 (TSA) (PRP)	Pseudoalteromonas haloplanktis (strain TAC 125)	22.115	-4.75659	9	0	+
PSHAa2977; B1199_19990	Q3IK06; A0A244CKW5	Secreted alkaline phosphatase; Alkaline phosphatase	Pseudoalteromonas haloplanktis (strain TAC 125); Pseudoalteromonas ulvae	56.104	-4.1791	10	2	+
glnA; glnA	A0A0A7EBM5; U1KVQ6	Glutamine synthetase	Pseudoalteromonas piratica; Pseudoalteromonas citrea DSM 8771	51.771	-3.10513	5	0	+
PP2015_904	A0A0S2K074	Flagellin	Pseudoalteromonas phenolica	33.272	-1.98111	8	6	+
A6E14_15830; aapJ	A0A1B9QUY2; A0A2N8ZA18	Amino acid ABC transporter substrate-binding protein; Amino-acid transporter subunit periplasmic-binding component of ABC superfamily	Vibrio genomosp. F10; Vibrio tapetis subsp. tapetis	36.779	-4.40352	9	3	+
PSHAa1824; SAMN02745724_03050	Q3IHE8; A0A1I1NQX5	Putative ferric enterobactin receptor; Iron complex outer membrane receptor protein	Pseudoalteromonas haloplanktis (strain TAC 125); Pseudoalteromonas denitrificans DSM 6059	108.47	-0.134083	1	6	-
BCU70_12530; JCM19236_807	A0A2N7D8W8; A0A0B8PY06	Oligopeptide ABC transporter substrate-binding protein OppA; Oligopeptide ABC transporter	Vibrio sp. 10N.286.49.C2; Vibrio sp. JCM 19236	60.845	-4.28191	5	0	+

Gene	Protein IDs	Fasta headers	Organism	Mol. weight [kDa]	Fold change[a]	Att. Peptides[b]	Vir. Peptides[b]	T-test[c]
VS_0814; JCM19238_4633	B7VKN2; A0A090P9S5	Flagellin	Vibrio tasmaniensis (strain LGP32); Vibrio ponticus	41.306	2.93242	0	6	+
PSHAa0108	Q3IDJ5	Putative TonB-dependent receptor putative outer membrane bound protein involved in iron chelated transport	Pseudoalteromonas haloplanktis (strain TAC 125	77.154	-0.386178	0	6	+
PSHAa0695	Q3IFM9	Putative outer membrane receptor for ferric iron uptake putative TonB-dependent receptor	Pseudoalteromonas haloplanktis (strain TAC 125)	78.238	-1.66131	0	5	+
rp1L; rp1L	Q3ILQ0; U1KUY2	50S ribosomal protein L7/L12	Pseudoalteromonas haloplanktis (strain TAC 125); Pseudoalteromonas citrea DSM 8771	12.23	-1.66069	6	5	+
PCIT_22080	U1KK96	Peptidase S8/S53 subtilisin kexin sedolisin	Pseudoalteromonas citrea DSM 8771	127.37	-3.70559	6	3	+
AKJ17_10950 ;VTAP4600_B0650	A0A0M0HMY3; A0A2N8ZK26	Maltoporin	Vibrio nereis; Vibrio tapetis subsp. tapetis	50.515	-0.600403	0	6	-
VS_II0677	B7VRS9	Ferrichrome-iron receptor	Vibrio tasmaniensis (strain LGP32)	78.505	-0.834848	0	5	-
fliC	Q3IDX4	Flagellin	Pseudoalteromonas haloplanktis (strain TAC 125)	28.504	0.124568	1	3	-
VPA0179; EA26_13395	Q87JR9;A0A099LYB7	Uncharacterized protein ; dTDP-glucose 4,6-dehydratase	Vibrio parahaemolyticus serotype O3:K6 (strain RIMD 2210633); Vibrio navarrensis	73.415	-3.83314	3	0	+

Gene	Protein IDs	Fasta headers	Organism	Mol. weight [kDa]	Fold change[a]	Att. Peptides[b]	Vir. Peptides[b]	T-test[c]
gcvH	B7VSK2	Glycine cleavage system H protein	Vibrio tasmaniensis (strain LGP32	13.88	-4.08161	3	0	+

[a] Fold change of virulent proteins related to attenuated proteins; [b] Razor and unique peptide counts; [c] Students t- test significance
